# The Human SLC7A5 (LAT1): The Intriguing Histidine/Large Neutral Amino Acid Transporter and Its Relevance to Human Health

**DOI:** 10.3389/fchem.2018.00243

**Published:** 2018-06-22

**Authors:** Mariafrancesca Scalise, Michele Galluccio, Lara Console, Lorena Pochini, Cesare Indiveri

**Affiliations:** ^1^Unit of Biochemistry and Molecular Biotechnology, Department DiBEST (Biologia, Ecologia, Scienze della Terra), University of Calabria, Rende, Italy; ^2^CNR Institute of Biomembranes, Bioenergetics and Molecular Biotechnology, Bari, Italy

**Keywords:** LAT1, SLC7 family, histidine, drug design, pro-drugs, proteoliposomes, molecular docking

## Abstract

SLC7A5, known as LAT1, belongs to the APC superfamily and forms a heterodimeric amino acid transporter interacting with the glycoprotein CD98 (SLC3A2) through a conserved disulfide. The complex is responsible for uptake of essential amino acids in crucial body districts such as placenta and blood brain barrier. LAT1/CD98 heterodimer has been studied over the years to unravel the transport mechanism and the role of each subunit. Studies conducted in intact cells demonstrated that LAT1/CD98 mediates a Na^+^ and pH independent antiport of amino acids. Some novel insights into the function of LAT1 derived from studies conducted in proteoliposomes reconstituted with the recombinant human LAT1. Using this experimental tool, it has been demonstrated that the preferred substrate is histidine and that CD98 is not required for transport being, plausibly, involved in routing LAT1 to the plasma membrane. Since a 3D structure of LAT1 is not available, homology models have been built on the basis of the AdiC transporter from *E.coli*. Crucial residues for substrate recognition and gating have been identified using a combined approach of bioinformatics and site-directed mutagenesis coupled to functional assays. Over the years, the interest around LAT1 increased because this transporter is involved in important human diseases such as neurological disorders and cancer. Therefore, LAT1 became an important pharmacological target together with other nutrient membrane transporters. Moving from knowledge on structure/function relationships, two cysteine residues, lying on the substrate binding site, have been exploited for designing thiol reacting covalent inhibitors. Some lead compounds have been characterized whose efficacy has been tested in a cancer cell line.

## Introduction

SLC7A5 is a transporter dedicated to essential amino acids. In the pre-genomic era, it was known as LAT1, an acronym standing for Large Amino Acid Transporter 1, that has endured over the time (Christensen, 1990). LAT1 belongs to the SLC7 family included in the larger APC (Amino acid-Polyamine-organo Cation) superfamily. The SLC7 family consists of 15 members, two of which are pseudogenes. The 13 encoded proteins are classified in two subgroups: the cationic amino acid transporters and the light subunits (LATs) of the heterodimeric amino acid transporters. Molecular evolution studies show that the heterodimeric amino acid transporters and the cationic amino acid transporters have a common ancestor characterized by 12 trans-membrane domains. This structure is conserved in the heterodimeric amino acid transporters, while the cationic amino acid transporters evolved to 14 trans-membrane domain structures, resulting from duplication of the last two trans-membrane domains, plausibly after branching of eukaryotes and archea (Verrey et al., 2004; Palacin et al., 2005; Fotiadis et al., 2013). The cationic amino acid transporters are N-glycosylated membrane proteins and are responsible for transport of cationic amino acids in cells. The heterodimeric amino acid transporters are present only in eukaryotes and are characterized by a broader substrate specificity toward neutral amino acids (SLC7A5, A8, A10, A12), aromatic amino acids (SLC7A15), negatively charged amino acids (SLC7A11) and cationic amino acids plus neutral amino acids (SLC7A6, A7, A9) (Fotiadis et al., 2013) (and refs herein). The structural peculiarity of the heterodimeric amino acid transporters is that of being one of the few examples of transporters composed by two different subunits: the light subunit, LATs and the heavy subunit, i.e., a membrane glycoprotein belonging to the SLC3 family with a single transmembrane domain and a large extracellular domain. The mentioned interaction is well conserved through evolution occurring via a disulfide bridge between two cysteine residues of the proteins forming heterodimeric amino acid transporters (Bröer and Brookes, 2001; Wagner et al., 2001; Palacín and Kanai, 2004). It is interesting to note that the SLC3 family, which comprises only two members (SLC3A1 and SLC3A2), is included in the SLC classification even if the direct involvement of these proteins in transport is not proven. In the case of LAT1, the heavy subunit counterpart is the SLC3A2, also known as CD98 or 4F2hc. The 3D structure of the ectodomain of the human CD98 is solved (Fort et al., 2007). The mentioned interaction is described since the early LAT1 discovery in rat glioma (Kanai et al., 1998). Several studies describe the function/structure relationships of such intriguing molecular organization, including the human isoform isolated and cloned in 1999 (Prasad et al., 1999; Fotiadis et al., 2013 and refs herein). In this frame, the term “LAT1” is often used to indicate the heterodimer LAT1/CD98, as well. In the sake of clarity, in this review we will use the term “LAT1” or “CD98” to indicate each of the monomers; we will use the term “LAT1/CD98” to indicate the heterodimer. It is important to stress that LAT1 is a key protein in cell growth and development due to its involvement in distribution of eight out of the nine essential amino acids to specific body districts such as placenta and Blood Brain Barrier (BBB, see section Gene and Tissue Localization of LAT1 and CD98). On the contrary, due to its low expression level in intestine and to its relatively low transport capacity, LAT1 is not responsible for absorption of amino acids from diet. This function is mediated by other high capacity transport systems located in the microvilli brush-border (Bröer and Bröer, 2017). An important evidence of the crucial role of LAT1 in cell metabolism and growth derives from the lethal phenotype of knockout animal embryo, which cannot go beyond the mid-gestation stage (E11.5), i.e., when nervous cells start to differentiate (Ohgaki et al., 2017). Over the years, the interest around this transporter moved from biochemical to bio-medical issues due to its involvement in important diseases such as cancer and neurological disorders (Fuchs and Bode, 2005; del Amo et al., 2008). In fact, the number of clinical studies, reporting LAT1 alterations in human pathology, is continuously growing, even though the molecular bases of these phenomena are still far from being completely deciphered. During the last years, important metabolic and signaling clues are emerging, thanks to a better characterization of human LAT1 (see following sections). In particular, targeting of human LAT1 becomes a hot topic in drug discovery for advancing the treatment of diseases in which the protein is involved.

An overview of *status artis* on this transporter will be provided in the present review, shedding light on the “LATest” findings.

## Gene and tissue localization of LAT1 and CD98

**Figure 1:**
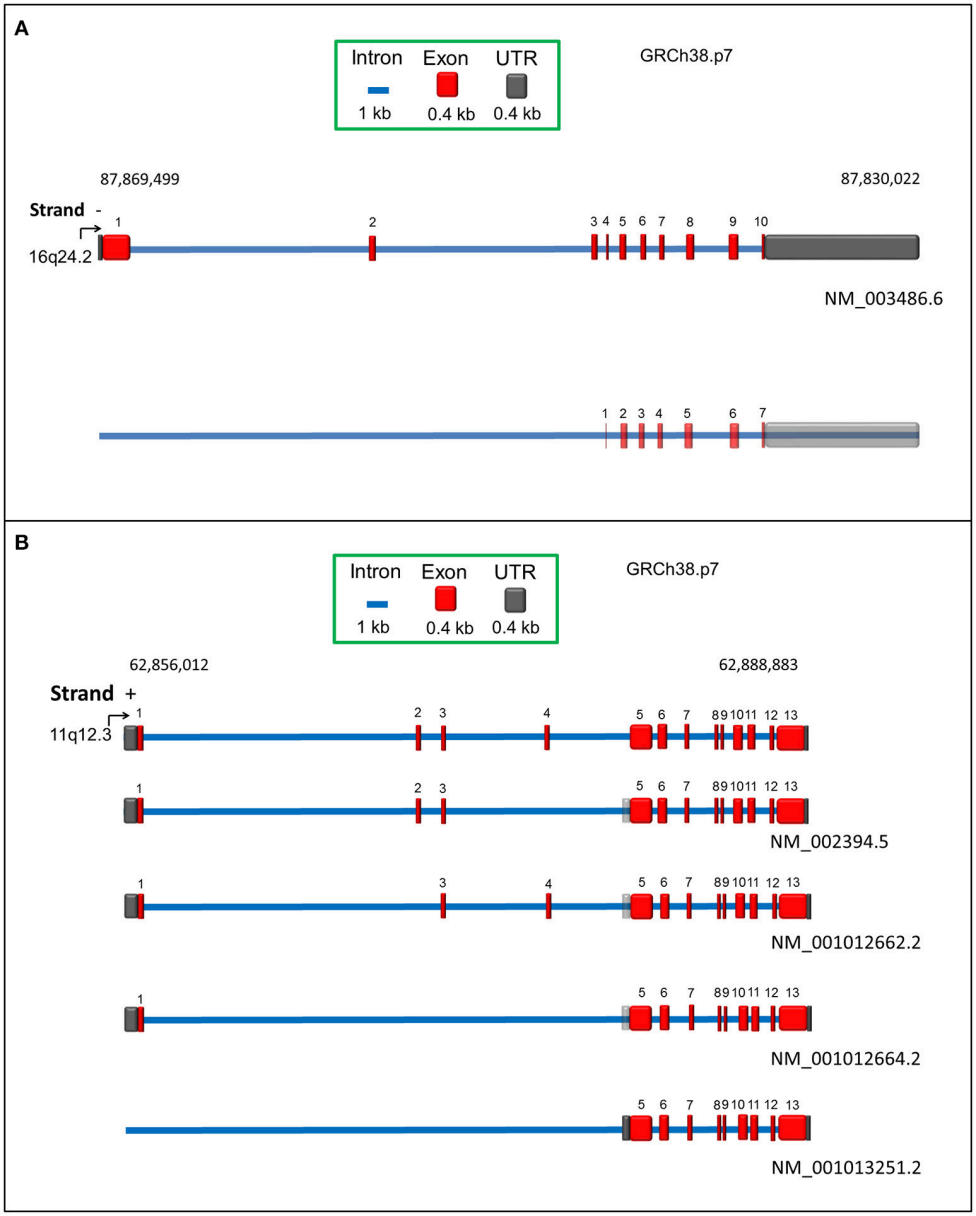
Schematic representation of human SLC7A5 **(A)** and SLC3A2 **(B)** genes according to RCh38.p7 genome assembly. Intronic and exonic sequences are depicted in blue and red, respectively. UTR sequence is indicated in dark gray. Predicted UTR sequences or isoforms are in transparency. For each transcript, the relative Genbank accession number is indicated.

The SLC7A5 gene, located at 16q24.2 (locus ID 8140), counts 39477 nucleotides with 10 exons (Figure 1A). Orthologs of this gene are present in 222 different organisms (https://www.ncbi.nlm.nih.gov/gene/8140). Two transcripts are reported in Ensemble (Figure 1A). One of these transcripts, NM_003486.6, codes for a protein of 507 amino acids, with a molecular mass of 55,010 Da. The other transcript derives from alternative splicing, but no evidence of a coded LAT1 protein is available, so far. Additional four transcripts are reported on NCBI databases, which, however, are only predicted. According to human protein Atlas project, RNA coding for SLC7A5 is ubiquitously expressed in all 27 tested tissues, even if at low levels (Fagerberg et al., 2014). Highest expression is measured in testis, bone marrow, brain and placenta (Prasad et al., 1999; Fotiadis et al., 2013). In polarized epithelia, LAT1 protein is mainly localized in basolateral membranes (Verrey et al., 2004; Fotiadis et al., 2013), with the exception of BBB where it is localized on both apical and basolateral membranes (Duelli et al., 2000). In placenta, LAT1 is on both maternal and fetal surfaces of syncytiotrophoblasts (Ohgaki et al., 2017). LAT1/CD98 heterodimer is also located in lysosomal membrane of HeLa cells (Milkereit et al., 2015).

The SLC3A2 gene, located at 11q12.3 (locus ID 6520), counts 32871 nucleotides with 13 exons (Figure 1B). Orthologous of this gene are present in 164 different organisms (https://www.ncbi.nlm.nih.gov/gene/6520). Four different transcripts are reported in Genbank database, coding for CD98 isoforms. The canonical isoform, NM_002394.5 (Figure 1B), results from 12 exons (exon 4 is not present) with total 2347 bp and encoding a protein of 630 amino acids, with a molecular mass of 67,994 Da. A three nucleotide longer isoform is also described, NM_001012662.2, which derives from an alternative combination of 12 exons, i.e., the presence of exon 4 but not of exon 2 (Figure 1B). These little variations at transcriptional level generate proteins with 95% identity and 1 amino acid length difference, being the second isoform, 631 amino acids long. The third transcript, NM_001012664.2, results from transcription of 10 exons, lacking exons 2, 3 and 4, counts 2161 nucleotides and codes for a protein of 568 amino acids. The last transcript, NM_001013251.2 differs in the 5′ UTR and lacks the first four exons, counting 1938 nucleotides: the corresponding protein, composed by 529 amino acids, derives from translation starting at a downstream ATG (Figure 1B). According to human protein Atlas project (Fagerberg et al., 2014), RNA coding for CD98 is ubiquitously detected with the highest expression level in kidney, placenta, testis and bone marrow. Expression of CD98 correlates with that of LAT1 in terms of localization, as expected from the interaction between the two proteins. However, CD98 is also expressed in other tissues since it works as ancillary protein of other SLC7 members, as well (Fotiadis et al., 2013 and refs herein).

## Function and substrate specificity: the double face of LAT1

**Figure 2:**
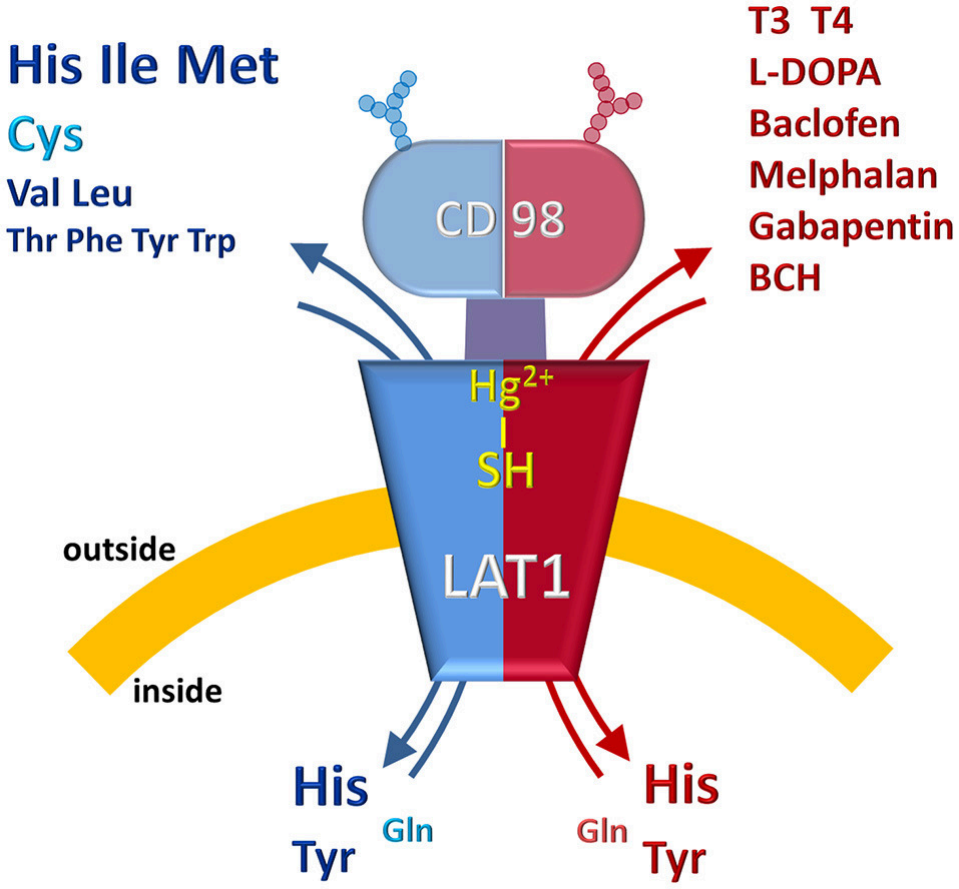
Representation of LAT1/CD98 and its substrates. The protein heterodimer is asymmetrically inserted in the cell membrane. The double-face feature of LAT1 is indicated by two-color design of the model. The blue half represents the specificity toward amino acids which are colored in blue (Essential Amino Acids) and light blue (Non-Essential Amino Acids). The size is suggestive of the higher or the lower specificity toward the amino acids. The red half represents the known specificity toward non-amino acid substrates: hormones, drugs and inhibitors. The reactivity of –SH group(s) of the protein toward Hg^2+^ is also represented. Glycosylation of CD98 is depicted as “antennas”.

**Figure 3:**
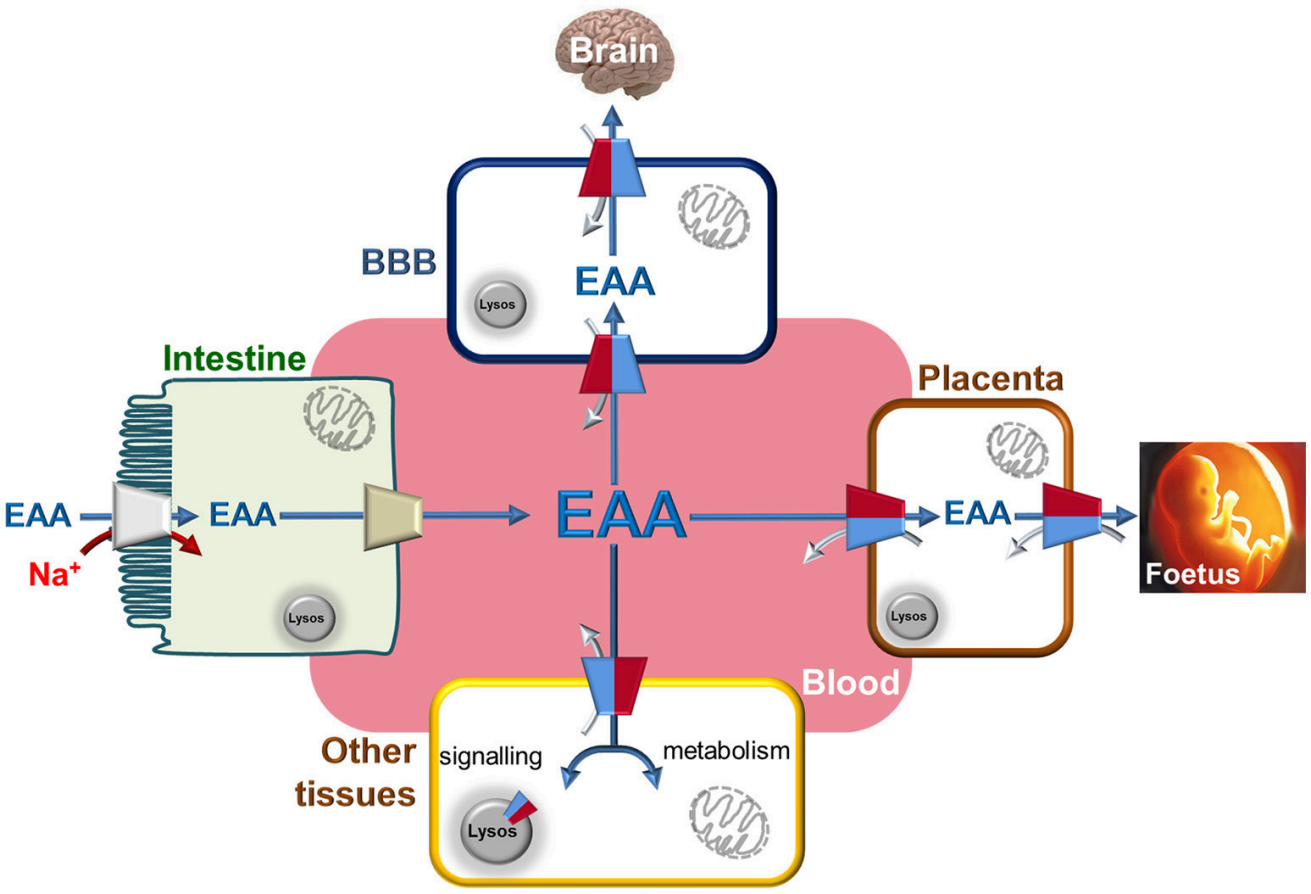
The Essential Amino Acids distribution network through LAT1. Interplay among blood and epithelial polarized cells of intestine (apical membrane depicted as brush-border and basolateral membrane in contact with blood), placenta, BBB (Blood Brain Barrier) and other tissues. Essential Amino Acids are absorbed through intestine using Na^+^ dependent transporters (indicated in gray) to allow their accumulation with high capacity. The same amino acids are conveyed to blood by other transporters present in the basolateral membrane of gut cells (indicated in light brown). From blood, Essential Amino Acids are accumulated in BBB and in placenta cells using LAT1 with an antiport reaction. In these cells, LAT1 is localized at both sides of cells allowing the flux of amino acids to Brain and Fetus, respectively. In other tissues, LAT1 is not highly expressed but plays the role of distributing Essential Amino Acids used both for signaling and metabolic purposes thanks also to alternative localization in lysosome membrane. These aspects became more important in tissues where LAT1 expression is strongly increased as in case of cancers. LAT1 is depicted as in Figure 2 by two-colored half. Flux of Essential Amino Acids are indicated by blue arrows (from blood to tissues) and by gray arrows (from tissues to blood).

The pioneer studies on LAT1/CD98 heterodimer are conducted in cell systems (such as *X. laevis* oocytes) measuring the uptake of essential amino acids using murine and human isoforms (Kanai et al., 1998; Mastroberardino et al., 1998; Prasad et al., 1999; Yanagida et al., 2001; Kim et al., 2002). These experiments establish that the transporter mediates an obligatory pH and Na^+^ independent antiport of tryptophan, phenylalanine, leucine and histidine with high affinity (Km for human isoform ranging from 5 to 50 μM) (Figure 2). The Na^+^ independence explains the relatively low transport capacity of this transporter and is in line with the low expression in the absorbent epithelia of intestine where, indeed, other transporters driven by Na^+^ gradient, such as SNATs, ATB^0, +^ and B^0^AT1, guarantee a massive uptake of amino acids (Pochini et al., 2014; Bröer and Bröer, 2017) (Figure 3). The heterodimer is able to recognize much less efficiently also glutamine (Km in the mM range); while, alanine, proline and charged amino acids are not recognized as substrates (Kanai et al., 1998; Mastroberardino et al., 1998; Kim et al., 2002; Meier et al., 2002; del Amo et al., 2008). Several reports show that the non-metabolizable analog BCH is a substrate of LAT1/CD98 (Figure 2) (Mastroberardino et al., 1998; Prasad et al., 1999; Kim et al., 2002). LAT1/CD98 also catalyzes the transport of the thyroid hormones T3 and T4 (Friesema et al., 2001; del Amo et al., 2008), of the dopamine precursor L-DOPA as well as of amino acid-related exogenous compounds, such as the drugs melphalan, baclofen and gabapentin (Uchino et al., 2002; del Amo et al., 2008) (Figure 2). The Vmax of L-DOPA transport is reduced upon depletion of plasma membrane cholesterol by methyl-β-cyclodextrin (Dickens et al., 2017). The ability of the transporter to accept canonical amino acids and other substances with some basic features of amino acids (Table 1), awards LAT1 with a two-faced role in physiological and in pathological contexts (Figure 2). Several controversies, however, are unsolved concerning the specificity issue due to some technical restrictions in the studies performed with intact cell systems. Thus, a novel drive derived from the availability of the recombinant human LAT1 and CD98 over-expressed in *E.coli* (Galluccio et al., 2013). The two human proteins were purified in a large scale and functionally characterized in the *in vitro* model of proteoliposomes. This is a versatile experimental tool, constituted by phospholipid vesicles, which allows the operator to precisely control both the external and internal spaces (Scalise et al., 2013). This triggered clarification of a key point of human LAT1/CD98 physiology: LAT1 is the sole transport competent subunit of the heterodimer, able to mediate antiport of amino acids with the same properties of the heterodimer, while CD98 does not exhibit any intrinsic transport function (Napolitano et al., 2015). Moreover, the transport protein is inserted in the proteoliposomal membrane with the same orientation as in the cell membrane: this represents an important requisite for translating the results obtained *in vitro*, to a physiological context. In the same system, the preference of LAT1 for amino acids has been definitively assessed (Figure 2). Functional and kinetic asymmetry of the transporter has been demonstrated (Napolitano et al., 2015): histidine and tyrosine are the bidirectionally-transported substrates, while the others are preferentially inwardly transported and glutamine is a poor substrate. Proteoliposomes harboring hLAT1 revealed suitable for studying the effect of mercury compounds: both the inorganic (HgCl_2_) and the organic (Methyl-Hg) forms of mercury strongly inhibit LAT1 mediated transport by binding to cysteine residue(s), in line with previous report on rabbit LAT1 (Boado et al., 2005; Napolitano et al., 2017a) (Figure 2). The results obtained with the purified protein substantiated the major role of LAT1 in histidine transport that, indeed, is already disclosed in the first important studies but then disregarded (Kanai et al., 1998; Mastroberardino et al., 1998; Prasad et al., 1999; Yanagida et al., 2001; Kim et al., 2002). The identification of histidine as LAT1 high affinity substrate *in vitro*, lays the groundwork for interpreting following important advances in human health (see section Regulation of LAT1 Expression). Histidine is, indeed, an essential amino acid eventually involved in aspartate and glutamate synthesis as well as in histamine production, thus being relevant for brain homeostasis and inflammatory response (Hasegawa et al., 2012; Sasahara et al., 2015). Interestingly, there is a concentration gradient for histidine across plasma membrane, being the plasma concentration of histidine one order of magnitude lower than the tissue concentration (Schmid et al., 1977). This gradient may constitute the driving force for extruding histidine in exchange with other essential amino acid such as leucine that becomes an important metabolite in pathological conditions underlying LAT1 function (see section LAT1 and Diseases). Altogether, the studies on specificity of hLAT1 for substrates and their analogs, as well as the interaction with non-transported molecules, allow extrapolating the essential features for a molecule to plausibly be transported by hLAT1: vicinal carboxylic and amino groups are essential, as resumed in Table 1. Indeed, dopamine, serotonine and GABA, which lack of vicinal carboxylic or the amino groups are not transported (Tarlungeanu et al., 2016). Furthermore, a large side group is also important, validating the pioneering acronym Large Amino Acid Transporter 1 (LAT1). Moreover, hydrophobicity seems an additional requirement for the side group, since, besides small, also charged amino acids are not transported. These features are already hypothesized in preliminary studies conducted in intact cells using the rat isoform of LAT1 (Uchino et al., 2002). Importantly, the main properties described *in vitro* are also confirmed by studies in intact cells both in the presence and in the absence of the disulfide between C164 of hLAT1 and C109 of hCD98 (Figure 2), demonstrating that CD98 is not required for intrinsic transport activity of LAT1 neither for substrate specificity (Pfeiffer et al., 1998; Campbell and Thompson, 2001; Boado et al., 2005; Napolitano et al., 2015). Therefore, it can be argued that the most probable function of the ancillary subunit is that of trafficking LAT1 to its definitive location in plasma membrane as suggested for both murine and human isoforms (Nakamura et al., 1999; Wagner et al., 2001; Franca et al., 2005; Cormerais et al., 2016). The role of CD98 is described in the case of SLC7A8 (LAT2). Differently from LAT1, LAT2 needs CD98 for both purification and stabilization of the protein, for functional and structural studies (Rosell et al., 2014). However, additional studies are required to ascertain the regulation of LAT1 function and the molecular mechanism of trafficking.

**Table 1:** The basic requirements for substrates of LAT1.

**Substrate**	**Structure**	**References**
HIS	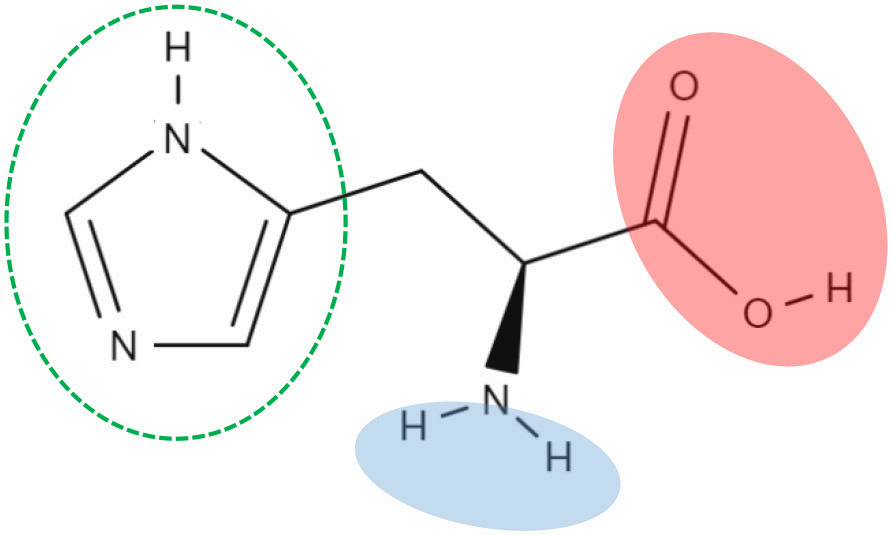	Mastroberardino et al., 1998; Napolitano et al., 2015
T3	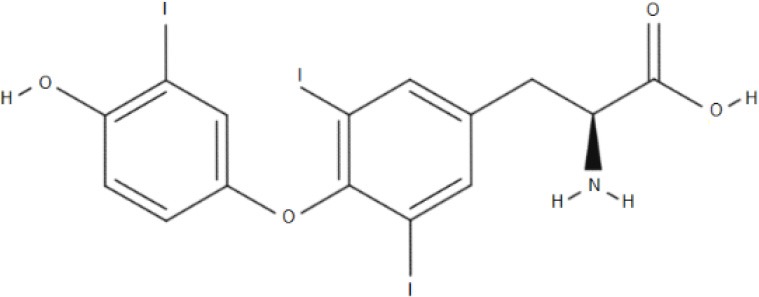	Friesema et al., 2001
T4	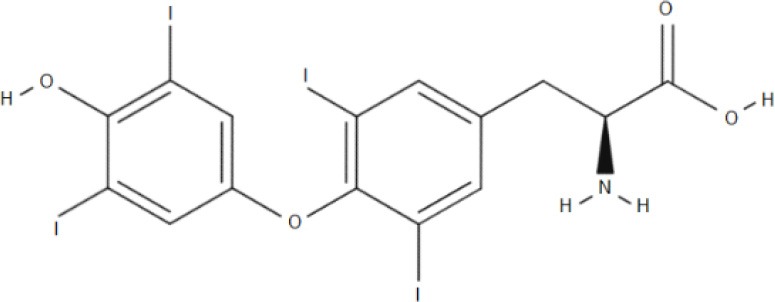	Friesema et al., 2001
L-DOPA	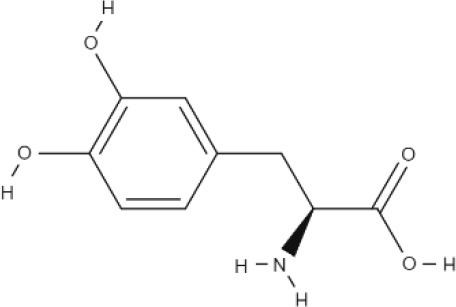	Kageyama et al., 2000
Baclofen	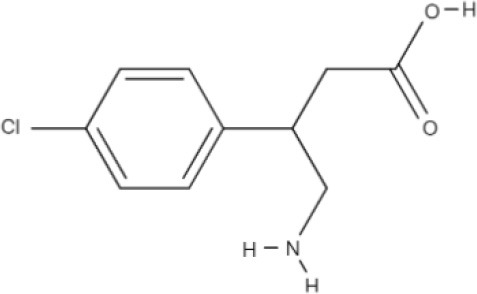	del Amo et al., 2008
Melphalan	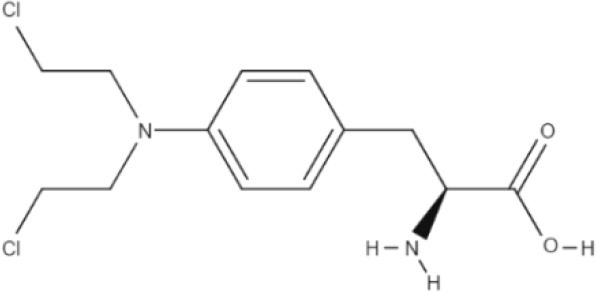	del Amo et al., 2008
Gabapentin	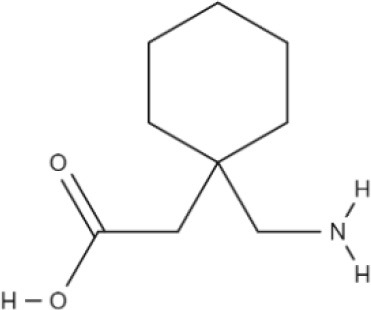	del Amo et al., 2008
BCH	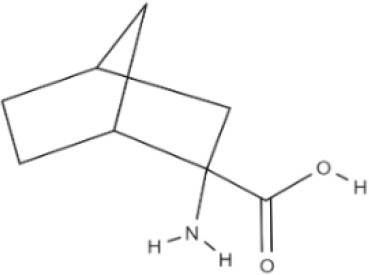	Mastroberardino et al., 1998

*Table lists the structure of known LAT1 substrates; on the histidine structure, the carboxylic group is highlighted by red circle, the amino group by blue circle, the hydrophobic portion by dotted green line. The same groups are present in all the structures below*.

## Kinetics and molecular mechanisms of LAT1

Similarly to other human plasma membrane transporters, the 3D structure of LAT1 is not available. Homology models are built on the basis of the bacterial homolog AdiC from *E.coli* (Geier et al., 2013; Napolitano et al., 2017a). This is an arginine/agmatine transporter characterized by a LeuT-fold (Gao et al., 2010; Ilgü et al., 2016) and represents a paradigm structure for the APC superfamily. However, the sole model does not allow drawing conclusions on the kinetic mechanism of transport and the molecular determinants of substrate recognition. To achieve this information, additional experimental approaches have been used in combination with bioinformatics. In this respect, the functionally active human recombinant protein has been employed in experiments conducted using proteoliposomes. Bi-substrate analysis, which could not be performed in intact cells, demonstrated that the amino acid antiport mediated by LAT1 occurs by a random simultaneous mechanism, i.e., with no preferential binding order of the substrates on the two opposed sides of the transporter. The collected data suggested that the internal and external substrates are translocated in a single transport cycle, implying the simultaneous exposure of binding sites for external and internal substrates (Napolitano et al., 2017a). This condition can be explained by the formation of functional homodimer whose existence has been proven by biochemical methodologies such as mild denaturing PAGE and cross-link approaches (Napolitano et al., 2017a). Noteworthy, this is in line with the oligomeric structure of the bacterial homologous AdiC (Gao et al., 2010) while it seems different from the data obtained with the LAT2/CD98 heterodimer that does not form homodimers (Rosell et al., 2014). Regarding the key determinants of the substrate binding site, some crucial residues that corresponded to those involved in gating of AdiC are predicted in previous study (Geier et al., 2013). These residues are F252, S342, and C407 in the human LAT1. More recently, using a combination of *in silico* methodologies, site directed mutagenesis and transport assay in proteoliposomes, we validated the predictions and identified the additional C335 residue as responsible for the interaction with the substrate (Napolitano et al., 2017a). Altogether, the combined approaches highlight that: (i) F252 plays the role of substrate gate opening and its aromaticity is essential to accomplish this function; (ii) S342 and C335 are crucial for histidine binding from the external side of the protein; (iii) C407 is, on the other hand, involved in substrate binding from the internal side (Napolitano et al., 2017a).

## Regulation of LAT1 expression

Notwithstanding the importance of LAT1 in mediating traffic of essential amino acids in both physiological and pathological conditions, little is known about regulation of its expression. In good agreement with the level of LAT1 protein in activated T lymphocytes, the cytokine IL-2 is able to up-regulate LAT1 expression (Sinclair et al., 2013). In rheumatoid arthritis, IL-17 is responsible for promoting LAT1-mediated migration of fibroblasts (Yu et al., 2018). Regulation by micro RNA (Miko et al., 2011) and long non-coding RNA is also reported (Yu et al., 2018). Moreover, DNA methylation occurring at promoter region seems to play a role in the regulation of LAT1 gene expression in human placenta across gestation (Simner et al., 2017). Glucose and insulin also modulate LAT1 expression: increase of glucose in diabetes induces a down-regulation of LAT1 expression with consequent sarcopenia in diabetes patients (Yamamoto et al., 2017). Conversely, glucose deprivation induces up-regulation of LAT1 in retina (Matsuyama et al., 2012). Interestingly, low insulin concentrations, upregulate LAT1 expression in muscle following mTORC1 activation (Walker et al., 2014). Vice versa, low expression of LAT1 in β-cells induces a strong reduction of insulin which is a protein constituted in large majority by the amino acids transported by LAT1 (Kobayashi et al., 2018).

The well documented over-expression of LAT1 in cancer is also explained by the presence, in the promoter region, of a canonical binding site for the proto-oncogene c-Myc (Hayashi et al., 2012) that, interestingly, regulates glucose metabolism (Kim et al., 2004). These evidences, even if fragmentary, suggest that a coordinate regulation of glucose and amino acid metabolism in cells may exist under both physiological and pathological conditions being in good agreement with the increased demand and transport of these nutrients in cancer (see section LAT1 and Diseases). Another pathway inducing expression of this transporter is mediated by YAP/TAZ, two transcriptional regulators which promote cell proliferation (Hansen et al., 2015). In renal carcinoma, LAT1 expression is increased by the hypoxia-inducible factor HIF2α that binds to LAT1 promoter (Elorza et al., 2012). In lung cancer, the activation of aryl hydrocarbon receptor pathway by diesel exhaust particles induces LAT1 up-regulation (Le Vee et al., 2016). It is also reported that in lung cancer LAT1 gives rise to a regulatory loop with the methyl transferase EZH2, involving the LAT1 negatively regulator RXRα, to control the methylation status of genes responsible for cell differentiation (Dann et al., 2015). In summary, from the mentioned works, a common denominator for regulation of LAT1 cannot be inferred at this stage. Anyway, the over-expression of this transporter in cancer cells refers to an increase of the protein amount and, hence, to an increased function as it occurs for glucose transporters (Ganapathy et al., 2009). Additional studies are needed to achieve a complete picture of the regulatory pathways involved in the control of LAT1 under physiological and pathological conditions.

## LAT1 and diseases

The link of LAT1 with cancer is nowadays well assessed. Indeed, over-expression of LAT1 is described in many human cancers and it certainly relates to metabolic changes occurring in cancer development and progression. In fact, transformed and malignant cells have specific metabolic requirements, which are collectively known as “Warburg effect” even if, over the years, this theory has been updated. A peculiar feature of cancer cells is the increased demand for nutrients such as glucose, essential amino acids and also glutamine, that becomes conditionally essential, for protein synthesis and/or energy supply (Ganapathy et al., 2009; Vander Heiden et al., 2009; Bhutia and Ganapathy, 2016; Scalise et al., 2017). Another hallmark of cancer cells is the requirement of leucine for mTOR activation in lysosome. Leucine is also a positive allosteric regulator of glutamate dehydrogenase in mitochondria, which, in turn, is responsible of glutamine fate (Scalise et al., 2017, and refs herein and see Figure 3). Thus, over-expression of LAT1 may respond to such specific needs together with other over-expressed amino acid transporters, such as ASCT2 and ATB^0, +^ (Pochini et al., 2014). In the past, a functional cycle involving LAT1 and ASCT2 has been suggested. In this cycle, glutamine, entered through ASCT2, may furnish the driving force for leucine uptake through LAT1 (Nicklin et al., 2009); this picture needs to be updated since glutamine cannot be an actual driving force for LAT1 being a very low affinity substrate. (Scalise et al., 2017). The important role of LAT1 in cancer is substantiated by the finding that this transporter is expressed in cancers of most human tissues, according to GENT database (Shin et al., 2011). LAT1 over-expression is also a prognostic factor of metastasis (Hayashi and Anzai, 2017). It is important to note that in most of the corresponding non-cancer human tissues, LAT1 is poorly expressed or, in some cases, absent (see section Gene and Tissue Localization of LAT1 and CD98). The ancillary protein CD98 is greatly over-expressed in cancers as well, according to human genome U133A array used for the creation of the GENT database (Shin et al., 2011). However, the over-expression of the two proteins is not inevitably linked because LAT1 mediates amino acid transport independently from CD98 (Napolitano et al., 2015) and CD98 is a protein with pleiotropic roles ranging from immune system regulation, cell growth activation, cell adhesion to integrin signaling (Chillarón et al., 2001; Palacín and Kanai, 2004; Cantor and Ginsberg, 2012; Fotiadis et al., 2013). Thus, its over-expression in cancer may have molecular basis different from those of LAT1. In this respect, CD98 is used disjointedly from LAT1, as target of antibodies raised for counteracting cell proliferation and metastasis (Behrens et al., 2015; Hayes et al., 2015). Besides cancer, LAT1 is involved in other diseases related to its expression in placenta and BBB (Figure 3). As example, LAT1 expression is reduced in Intra-Uterine Growth Restriction (IUGR) (Pantham et al., 2016). This is a high risk condition for perinatal complications, characterized by reduced concentrations of leucine and phenylalanine. Moreover, intra-uterine growth restriction increases the risk of developing cardiovascular and metabolic diseases in childhood and adulthood. Conversely, maternal obesity is a possible cause of up-regulation of placental LAT1 in mouse model with consequent fetal overgrowth (Rosario et al., 2015). This represents a high risk condition for insulin resistance at birth and for developing type 2 diabetes in childhood and adulthood. Regarding the BBB, a decreased expression of LAT1 in this district is linked to onset and development of Parkinsons's disease (Ohtsuki et al., 2010). The molecular basis of the link is the reduced distribution of the dopamine precursor, L-DOPA, which is transported by LAT1, besides that of essential amino acids (Kageyama et al., 2000). The role of LAT1 in brain is also explained by transport of tryptophan, which is important for normal neurological development (Asor et al., 2015). More recently, alteration of LAT1 function in BBB caused by two natural mutations, has been described as the molecular determinant of Autism Spectrum Disorders (ASD); it is important to highlight that histidine, among the LAT1 substrates, exhibited in brain the greatest variation of concentration in the pathological phenotype (Tarlungeanu et al., 2016). This correlates well with the biochemical identification of histidine as the highest affinity LAT1 substrate confirming its relevance in underlying the pathophysiological role of LAT1 (Napolitano et al., 2015). On the basis of the experimental data, the molecular mechanism responsible for the pathological phenotype has been revealed: in particular, the defective mutants of LAT1 are not able to perform the exchange transport reaction resulting in a net accumulation of histidine in brain and a lack of other essential branched chain amino acids (Tarlungeanu et al., 2016). Furthermore, LAT1 is involved in activation of T cells accompanied by metabolism enhancement (Hayashi et al., 2013). Finally, in inflammatory conditions, such as pancreatitis, LAT1 expression decreases (Rooman et al., 2013). This is linked to the function of LAT1 in acinar cells that allows accumulation of amino acids necessary to synthesize digestive enzymes (Rooman et al., 2013). Some previous works suggest that LAT1 can also mediate uptake of mercury compounds explaining their toxicity for fetal growth (Kajiwara et al., 1996; Bridges and Zalups, 2017); moreover, our inhibition studies by mercury compounds suggested that mercury-derivatives can impair essential amino acids transport in cells expressing LAT1 (Napolitano et al., 2017a). Also in BBB, the ability of LAT1 to mediate mercury compounds uptake may explain its toxicity in brain (Boado et al., 2005; Bridges and Zalups, 2017). Altogether, these findings highlight a crucial role of LAT1 in several human pathologies.

## Link of LAT1 with amino acid molecular sensors

Moving from the observations of tissue distribution and substrate specificity, it can be concluded that the physiological role of LAT1 consists in maintaining the concentration of essential amino acids, particularly in those body districts, such as brain and placenta (Figure 3), where these molecules are fundamental for normal growth and development (Fotiadis et al., 2013; Bröer and Bröer, 2017). Over the years, it became more and more clear that the same applies to cancers whose cells are addicted to amino acids (Ganapathy et al., 2009; Bhutia and Ganapathy, 2016).

All these features eventually merge into mTOR, a serine/threonine kinase belonging to the PI3K-related family (Milkereit et al., 2015; Cormerais et al., 2016; Saxton and Sabatini, 2017). In fact, genetic or chemical disruption of LAT1, but not CD98, triggers, on one hand, mTOR inhibition and, on the other, GCN2 activation enhancing amino acid stress response (Gallinetti et al., 2013; Cormerais et al., 2016). GCN2 is a serine/threonine kinase and together with mTOR are responsible for handling amino acids concentration in cells; their cooperation occurs with a different degree of cross talk depending on the stress conditions (Carroll et al., 2015).

The relationship between mTOR and LAT1 is a long lasting field of investigation also because mTOR hyper-activation is often described in cancers (Saxton and Sabatini, 2017; Wolfson and Sabatini, 2017). The network of proteins regulating and regulated by mTOR is very intricate and many efforts have been made over the years to dissect each player. The importance of amino acids in this scenario has been proposed very long ago, but only recently, the puzzle reached a quite complete form. Among the amino acids, glutamine, arginine and leucine are involved in activation of mTOR (Wang et al., 2015; Rebsamen and Superti-Furga, 2016). In this respect, the function of LAT1 is related to its ability in mediating uptake and accumulation of leucine in cells. The leucine taken up via LAT1, is sequestered by Sestrin2 causing an increase of free GATOR2 with consequent increase of mTOR signaling (Lee et al., 2010; Wolfson and Sabatini, 2017). Sestrin2, in fact, acts as a negative regulator of mTOR activity. Leucine sensed by Sestrin2 derives from plasma membrane uptake as well as by efflux from lysosomes. In this respect, it is worth of note that LAT1 mediates efflux of leucine from lysosomes as well (Figure 3) (Milkereit et al., 2015).

## LAT1 druggability and clinical outcomes

Given the above-described premises, it is not surprising that LAT1 is a relevant pharmacological target for several diseases. This protein is exploited for both drug delivery and chemical knocking-out, i.e., block of transport activity exerted by pharmacological compounds. The drug delivery issue is particularly relevant in the BBB due to the impermeability of this barrier to exogenous substances. This feature is responsible for inefficacy/low efficacy of several pharmacological treatments. Thus, the relatively wide substrate specificity of LAT1 is the pre-requisite for the “pro-drug” approach, searching for compounds fulfilling the requirement for transport competence (Table 1) and, hence, for crossing the BBB by a LAT1 mediated process (Peura et al., 2011; Zur et al., 2016). This strategy has the scope of improving pharmacodynamics of drugs targeting brain for neurological disorders (Puris et al., 2017). Several pro-drug compounds have been synthesized so far, whose chemical properties and, in some cases, delivery in specific tissues have been described. Among the compounds, LAT1 substrate derivatives of ketoprofene, valproate and perforin inhibitors seem to be efficiently delivered in model tissues (Gynther et al., 2008, 2010, 2016; Peura et al., 2011; Huttunen et al., 2016; Puris et al., 2017). Melphalan (Table 1 and Figure 2), which is already used as a chemotherapy agent, can also be considered a LAT1 based prodrug (Kim et al., 2002; Ganapathy et al., 2009) even if some contradictory findings are reported (Uchino et al., 2002; Nakanishi and Tamai, 2011). The over-expression of LAT1 in several type of human cancers and its ability to transport modified substrates allow to exploit this protein in diagnostics and clinics, PET (Positron Emission Tomography) and BNCT (Boron Neutron Capture Therapy), respectively. PET allows the diagnosis of tumors by tracing accumulation of radiolabeled molecules specifically in cancer foci. The used molecules are tyrosine, phenylalanine and methionine derivatives, which are delivered to cells via LAT1 (Hayashi and Anzai, 2017) (and refs herein). BNCT is, on the contrary, an emerging anticancer therapy based on fission reactions that occur when boron is irradiated with neutron beams (Wongthai et al., 2015; Hayashi and Anzai, 2017). The essential condition for BNCT to work is the accumulation of boron that is guaranteed by the use of boronophenylalanine, another substrate derivative of LAT1, which is the most effective (Wongthai et al., 2015). Together with the efforts devoted to improve drug delivery and therapy, LAT1 is object of several studies aimed to identify potent and specific inhibitors able to chemically knockout the over-expressed protein. This important task, however, cannot be exhaustively performed using the virtual drug design approach, owing to the absence of a 3D crystallographic structure of LAT1 (see section Function and Substrate Specificity: The Double Face of LAT1). Indeed, all the works published so far dealing with bioinformatics, are based on homology models and require some validation by parallel approaches (Fang et al., 2009; Gao et al., 2010; Kowalczyk et al., 2011; Napolitano et al., 2017a). Two main strategies are followed searching for inhibitors: competitive inhibitors or non-competitive inhibitors design. In the first case, substrate-mimicking molecules are obtained, able to interact with the substrate binding site of the protein. It is worth to note that the tyrosine analog JPH203, previously known as KYT-0353, is a potent inhibitor (Oda et al., 2010) both *in vitro* and in mouse model of HT-29 tumors (colon cancer) (Oda et al., 2010; Wempe et al., 2012; Toyoshima et al., 2013). The molecular mechanism of action of JPH203, investigated using osteosarcoma cell line (Choi et al., 2017), consists in activating the mitochondrial pro-apoptotic pathway. This inhibitor is effective also in different type of cancers (Rosilio et al., 2015; Hayashi et al., 2016; Choi et al., 2017; Otsuki et al., 2017; Yothaisong et al., 2017). Moreover, a synergistic effect with metformin is observed in a cell line of HNC (Head and Neck Cancer) *in vitro* and in mouse-transplanted model (Ueno et al., 2016). Other ligands are proposed by using integrated approach of virtual screening of drug libraries and in *in vitro*/*ex vivo* models using cis-inhibition and trans-stimulation assays: phenylalanine and tyrosine analogs (Geier et al., 2013; Augustyn et al., 2016), triiodothyronine (T3) analogs (Kongpracha et al., 2017), tryptophan analogs (Ylikangas et al., 2014) and hydroxamic acids conjugated to LAT1 substrates (Zur et al., 2016); in the mentioned works, IC50 values are measured ranging from 1 μM to more than 300 μM. The effect of these ligands on cell proliferation is also evaluated to give information on potential pharmacological efficacy. Interestingly, the substrate analog strategy is also followed for other transporters such as ASCT2, that represents another key target for development of new anti-cancer drugs (Pochini et al., 2014; Bhutia and Ganapathy, 2016). In this case, glutamine and serine analogs have been designed and tested in cancer cell lines for their ability of blocking ASCT2 transport activity as recently reviewed (Pochini et al., 2014; Scalise et al., 2017). However, the described approaches can have some frailty if the concentration of natural amino acid substrates increases displacing the inhibitor and, thus, leading to less efficient effects (Augustyn et al., 2016). Then, after identification of two cysteine residues, i.e., C335 and C407, in the substrate binding site of LAT1 (Napolitano et al., 2017a) (see section Kinetics and Molecular Mechanisms of LAT1) we tried to apply an alternative strategy, i.e., design of inhibitors that could bind covalently to the cysteine residues leading to non-competitive inhibition. This study has been conducted *in vitro* in proteoliposomes carrying hLAT1 and validated in a cancer cell line (Napolitano et al., 2017b). The tested compounds belong to the dithiazole group, characterized by a favorable reactivity toward thiol functional groups of cysteine. Interestingly, some dithiazole derivatives are already known for their anti-fungal, anti-microbial and anti-tumor activities (Konstantinova et al., 2009). The inhibitors displaying the most potent effects react with C407, as demonstrated by the loss of efficacy on the C407A mutant. The action of these inhibitors on LAT1 may be related to the dithiazole moiety, which resembles the imidazole ring of histidine. Thus, inhibitor's recognition could be ascribed also to a “substrate-like” structure, but the molecular mechanism of stable inhibition is based on the disulfide bond with C407. In this case, in fact, the presence of substrate does not displace the inhibitor from the interaction with LAT1. The experimental data were corroborated by *in silico*-based analysis on the homology model of LAT1 (Napolitano et al., 2017b).

## Conclusions

The relevance of LAT1 for human metabolism lies on the ability of this protein to recognize two classes of different substrates: essential amino acids and hormones as main physiological substrates and some drugs as non-physiological substrates. This two-faced feature elected LAT1 as a crossroad point in cell life. Indirect proof of such a statement is the occurrence of different kind of pathologies with wide degree of severity associated with alterations of function/expression of LAT1. The eminent position assumed by LAT1 is somewhat in contrast with the scarce depth of knowledge so far achieved. In fact, despite great efforts, which have been made to decipher the biology of LAT1, a complete scenario is not yet depicted. Combined approaches of bioinformatics, *in vitro* and *ex vivo* experimental approaches have been used to shed light on some dark sides of LAT1 transport mechanism, substrate specificity and regulation. These results gave a strong input to pharmacological studies, which led to identification of different classes of molecules able to interact with LAT1 either as non-transported inhibitors and as transported substrates with important outcome in human health. However, other biochemical aspects still need to be solved, such as the trafficking, the regulation of expression/function, and the effect of potential post-translational modifications on LAT1 stability/activity as well as the interaction with other transporters and enzymes. In fact, an integrated view of LAT1 activity in the cell context is necessary to completely understand the physiological role of this protein. An important issue regarding LAT1 and pharmacological outcomes is related to its peculiar expression in BBB, which is the physical barrier protecting the brain from xenobiotics but also the main route to provide this district with essential amino acids. In conclusion, the findings collectively derived from different works opened new perspectives also for translational medicine.

## Author contributions

MS contributed in collecting bibliography, preparing figures and writing; MG, LC, and LP contributed in drawing structure figures and writing; CI contributed in writing and supervision of all the activities.

### Conflict of interest statement

The authors declare that the research was conducted in the absence of any commercial or financial relationships that could be construed as a potential conflict of interest.

## Abbreviations

LAT1: Large Amino Acid Transporter 1
BBB: Blood Brain Barrier
BCH, 2-aminobicyclo-(2, 2: 1)-heptane-2-carboxylic acid
IL-2, IL-17, interleukin-2: interleukin- 17
mTOR: mammalian Target of Rapamicyn
GCN2: general control nonderepressible 2
PET: Positron Emission Tomography
BNCT: Boron Neutron Capture Therapy.
